# Closing the Loop: Building Data Systems That Inform and Improve Clinical Practice

**DOI:** 10.1111/ans.70331

**Published:** 2025-10-03

**Authors:** Magdalena Sejka, Christobel Saunders, Anita Skandarajah

**Affiliations:** ^1^ The University of Melbourne Melbourne Australia; ^2^ The Royal Melbourne Hospital Melbourne Australia

There is growing consensus that patient care should be evaluated by patient‐centered outcomes, and not just by metrics such as procedural volume or complication rates in the case of surgery, for example. Benchmarking frameworks such as those developed by the International Consortium for Health Outcomes Measurement (ICHOM) offer a rigorous foundation for measuring what matters—survival, function, quality of life, and equity [1]. Yet, in most Australian and New Zealand centers, these frameworks remain aspirational rather than operational. The primary barrier is not a lack of clinician interest, but a lack of infrastructure [2].

As part of an institutional exploration into the feasibility of adopting the ICHOM Breast Cancer Standard Set, we reviewed the availability of the required 123 data elements. These included tumor characteristics and treatment dates, complex fields like chemotherapy regimens, recurrence status, and comorbidities. The mapping process involved assessing two main sources: a hospital‐based database maintained by administrative and clinical staff and the electronic medical record (EMR) with a planned integration of patient‐reported outcome measures (PROMs).

Despite the EMR's (EPIC) sophistication, only 20% of required data were extractable in structured format; 58% required time‐intensive notes review. PROMs were not routinely collected. What emerged was not just a fragmented dataset, but a fragmented process, heavily reliant on manual effort. This was a feasibility review, not an implementation project; yet aligning routine care with outcome standards proved overwhelmingly complex, even in large, well‐resourced metropolitan hospitals with a mature EMR. This underscores the scale of the interoperability challenge across Australia and New Zealand, particularly in rural and private settings where EMRs often do not exist.

This experience is not unique. In Australia and New Zealand, data systems remain fragmented and rely heavily on unpaid clinician effort. Outcome benchmarking is often maintained by junior doctors, students, or clinicians working outside rostered hours. While this reflects professional commitment, such reliance is unsustainable. Embedding outcome measurement into routine care requires institutional responsibility. There are also ethical concerns: expecting doctors to support system‐level functions without formal support may contribute to inequity and burnout. Structured, resourced approaches are needed.

However, it is possible: the Australian and New Zealand Audit of Surgical Mortality captures close to 100% of inpatient surgical deaths, with peer‐reviewed audits driving improved care [3, 4]. This is possible because data submission is mandated, backed by structured processes, and integrated into hospital workflows—demonstrating the impact of coordinated governance.

The BreastSurgANZ Quality Audit (BQA) is a strong example of a specialty‐led initiative that has delivered consistent surgical audit data across Australasia [5]. Maintained through professional engagement, it plays an important role in credentialing and continuous professional development. As expectations evolve, BQA has the potential to expand its role in improving care. But without stronger structural support, such as alignment with EMRs, formal integration of PROMs, and governance that enables collaboration with international frameworks like ICHOM, this potential may not be realized. Investment in infrastructure and systems is essential for BQA to move from data collection to meaningful outcome benchmarking.

The Australian Breast Device Registry (ABDR) illustrates what can be achieved with formal support and system integration [6]. Funded by the Commonwealth Government and governed through a multidisciplinary steering committee, it monitors the safety and performance of breast implants and expanders at a national level. Through standardized reporting, surgeon feedback, and post‐market surveillance, ABDR demonstrates how structured, resourced data collection can influence both clinical practice and policy.

Internationally, Sweden's national quality registries and the Santeon initiative offer powerful examples. Sweden integrates clinical data, PROMs, and survival data across registries, achieving substantial returns in efficiency and safety [7]. In the Netherlands, Santeon hospitals use shared dashboards to benchmark cancer care and iteratively improve practice. After three quality improvement cycles in breast cancer, they reduced reoperation rates and increased day‐case surgery [8].

One crucial gap in Australian data systems is the lack of structured feedback loops.

Data are rarely fed back in real time or used systematically for quality improvement. For outcome measurement to drive change, information must inform practice across the micro (individual clinician), meso (department or hospital), and macro (policy or system) levels. NHS Wales has begun addressing this challenge, embedding outcome feedback mechanisms into routine workflows [9].

These successes reflect key enablers that are often missing in Australian systems:

*Barriers:*

- Data are unstructured and fragmented. EMRs are not built for outcome capture, and relevant data are often buried.
- Lack of dedicated personnel. Databases are usually maintained by individuals, not institutions, often lacking formal training.
- Clinicians face time and workflow constraints, limiting their ability to use data meaningfully.
- Outcome measurement is often seen as an “extra,” rather than integral to care.
- PROMs and PREMs are inconsistently collected and rarely integrated into clinical databases or EMRs.

*Solutions:*

- Data harmonization is a prerequisite for scalable outcome measurement. Mapping agreed outcome datasets to EMR fields replicated across hospitals.
- Design systems for usability and automation, making high‐quality data entry and follow‐up a natural part of care. This is particularly relevant to PROMs and PREMs collection, which requires sustained organizational commitment and resourcing, given the longitudinal nature and perceived misalignment with routine clinical workflows.
- Government‐funded initiatives aimed at building interoperable health data systems, shaped by clinicians.
- Fund and embed data managers, analysts, and informaticians within clinical teams.
- Allocate protected non‐clinical time for data‐driven quality improvement.
- Reframe outcome and experience measures as integral to clinical care, not administrative overhead.
- Health services research units can support this shift by expanding their role beyond traditional research to include benchmarking, data curation, and collaboration with clinical teams on quality improvement.
- Emerging AI technologies will assist in extracting structured data from EMRs, but still need human leads.

Our findings highlight the futility of outcome measurement efforts without all three elements of a learning health system: iterative improvement cycles, socio‐technical infrastructure to support them, and governance to sustain them [10]. Without these, efforts remain fragmented and dependent on goodwill rather than system responsibility.

## Conflicts of Interest

The authors declare no conflicts of interest.

## Data Availability

Data sharing not applicable to this article as no datasets were generated or analzsed during the current study.
